# Non-native Minnows Threaten Quillwort Populations in High Mountain Shallow Lakes

**DOI:** 10.3389/fpls.2018.00329

**Published:** 2018-03-22

**Authors:** Esperança Gacia, Teresa Buchaca, Nayeli Bernal-Mendoza, Ibor Sabás, Enric Ballesteros, Marc Ventura

**Affiliations:** ^1^Integrative Freshwater Ecology Group, Centre d'Estudis Avançats de Blanes (CEAB-CSIC), Blanes, Spain; ^2^Benthic Ecosystem Functioning Group, Centre d'Estudis Avançats de Blanes (CEAB-CSIC), Blanes, Spain

**Keywords:** minnows, quillworts, lakes, invasions, eutrophication, algae, epiphytes

## Abstract

Submersed aquatic plants are a key component of shallow, clear water lakes contributing to primary production and water quality. High mountain lakes are naturally fishless although invasive trout and most recently minnows have been introduced causing a major impact on fauna richness. The Pyrenean high mountain range has preserved soft-water oligotrophic boreal isoetids in their southern limit of distribution but the recent fish introduction is a potential factor of stress that needs to be addressed. We here work under the hypothesis that due to contrasting ecological features, trout will not be heavily affecting quillwort populations while minnows will have a stronger effect on zooplankton and zoobenthos that will promote algal growth and reduce light availability for the underwater meadows. Ten Pyrenean shallow lakes representative of three scenarios -fishless, with trout and with minnows-, were sampled for meadow structure, water column and benthic environment characterization in mid-summer 2015 and 2016. Quillwort biomass allocation (above vs. belowground), epiphytic load, and composition of the algal community (abundant cyanobacteria) differed in the presence of minnows. In trout lakes biomass allocation and epiphytic load were average and the algal community composed by chlorophytes and diatoms as in fishless lakes. Biomass ratio was close to thresholds of negative buoyancy in minnow lakes indicating that meadows were at risk of uprooting and consequent de-vegetation. Total and soluble carbohydrates were lower and the sporangia contained significantly less reserves to constrain growth and expansion in the presence of minnows. Lake scenarios were coupled to physicochemical differences with low light, high phosphorus and Chl-a (mesotrophia) in minnow lakes, while trout and fishless lakes remained oligotrophic. This is the first study assessing the impact of non-native fish on soft-water isoetids from mountain lakes and shows that minnows are a major threat to quillworts. The impaired light environment (from epiphytic algal overgrow and water column Chl-a) entails consequent regression (i.e., no recruitment) and de-vegetation (uprooting) of the meadows. Since soft-water oligotrophic mountain lakes are protected under the Habitats Directive, some action needs to be urgently implemented not only to preserve quillworts but to the overall ecological integrity of the lakes.

## Introduction

Aquatic macrophytes are key structural elements in non-altered shallow lakes and ponds were they provide shelter to associated fauna and modulate trophic interactions (Roth et al., 2007). Submersed aquatic plants are threatened by eutrophication, a process that fosters fast growing algae (phytoplankton and epiphytes) against more structured macrophytes as a result of nutrient increase. Eutrophication causes a deterioration of the light environment (Scheffer et al., 2001; Scheffer and Carpenter, 2003) and increases chances of submersed plant mortality. Trophic characteristics of clear and turbid phases are well known; high macrophyte cover, high piscivorous vs. planktivorous fish and high zooplankton vs. phytoplankton ratios are found in clear lakes (low water column chlorophyll) while opposite tendencies with high mid-summer water column chlorophyll, and low to nil macrophyte biomass and production are encountered in turbid lakes (Meerhoff and Jeppesen, 2009).

The shift from a clear to a turbid phase in a lake can be driven by non-native species. For instance, invasive crayfish can increase bare sediment cover by overgrazing aquatic macrophytes, which promotes phosphorus release and subsequent eutrophication (Marchi et al., 2011; Arribas et al., 2014). Other invasions threaten submersed macrophytes when impacting trophic groups (i.e., macro-invertebrates) that modulate epiphyte-macrophyte competence for light and nutrients (Jones and Sayer, 2003).

High mountain lakes are usually isolated from lower streams by physical barriers that have prevented natural colonization of fish (Knapp et al., 2001; Miró and Ventura, 2013). Fish introductions, however, add a top predatory level to the trophic structure of the lake, which can lead to profound ecological changes (Ventura et al., 2017). The introduction of salmonids in fishless lakes is commonly associated with extirpation or reduction of native aquatic species (e.g., invertebrates and amphibians) and can have indirect effects on the whole ecosystem (Knapp et al., 2001; Eby et al., 2006; Tiberti et al., 2014). The introduction of minnows has been shown to have a strong impact on zooplankton biomass and size distribution (Schabetsberger et al., 1995). However, even if different studies have reported effects of fish introduction on different high-mountain and north-temperate lake ecosystem compartments (other fish populations, invertebrates, and plankton), its effect on high mountain lake macrophytes has been surprisingly neglected.

The Pyrenean high mountain range shelters thousand oligotrophic (nutrient poor) soft-water (weakly buffered) lakes with good water and ecosystem quality (Catalan et al., 1993; Camarero and Catalan, 2012). Some of those lakes shelter dense and diverse macrophytic communities including isoetids (Gacia et al., 1994; Chappuis et al., 2011, 2014). Those are slow growing aquatic plants with stiff leaves or stems forming basal rosettes. The functional group receives the name after the genus *Isoetes* such as the species studied here (*I. lacustris*) that is known as quillwort. In Pyrenean lakes, communities with isoetids have maintained macrophyte biodiversity (Alahuhta et al., 2017) and are reference sites for soft-water oligotrophic boreal species such as *Isoetes lacustris* L., *Isoetes echinospora* L., and *Subularia aquatica* L., which are currently threatened in Northern and Central Europe (Sand-Jensen et al., 2000; Arts, 2002; Pulido et al., 2012).

In the Pyrenean lakes first fish (*Salmo trutta*) introductions date back to the fourteenth and fifteenth centuries (Miró and Ventura, 2013), although most salmonid introductions (with *S. trutta* and also *Oncorhynchus mykiss* and *Salvelinus fontinalis*) took place during the second half of the twentieth century, being present nowadays in more than half of the lakes (Miró and Ventura, 2013). Minnows (*Phoxinus* sp.) have been recently introduced associated to their use as live bait for salmonids (Miró and Ventura, 2015).

In this study we search for traces of fish invasions on aquatic macrophyte community performance in Pyrenean shallow lakes and specifically on meadows of the perennial quillwort *Isoetes lacustris* L., which is a common and abundant macrophyte at altitude ranges from 1,800 to 2,400 m a.s.l. (Gacia et al., 1994; Chappuis et al., 2011). The species has a major ecological role in lake functioning by promoting sediment oxygenation (Smolders et al., 2002; Gacia et al., 2009), denitrification (Vila-Costa et al., 2016) and phosphorus immobilization (Jones et al., 1994). It also shows high resilience in front of perturbations such as moderate water level fluctuations (Rørslett, 1989) but its recovery is extremely slow (recent estimates of >60 years; Riera et al., 2017) and therefore we urge to search for potential early indicators of stress associated to introduced fishes.

Here we will be comparing quillwort population indicators of stress in shallow lakes of the same glacial origin and altitude but exposed to different scenarios: fishless (i.e., natural), with trout, and with minnows. Our hypothesis is that fish will modify the lake environment to bounce on isoetid populations. We also expect the effect to be different depending on whether the lake has trout or minnows due to their contrasting ecological features. Minnows will likely have a stronger effect on zooplankton and zoobenthos and therefore promote algal blooms that will decrease light availability thus potentially affecting the viability of quillwort populations.

## Methods

### Field work

**Figure 1:**
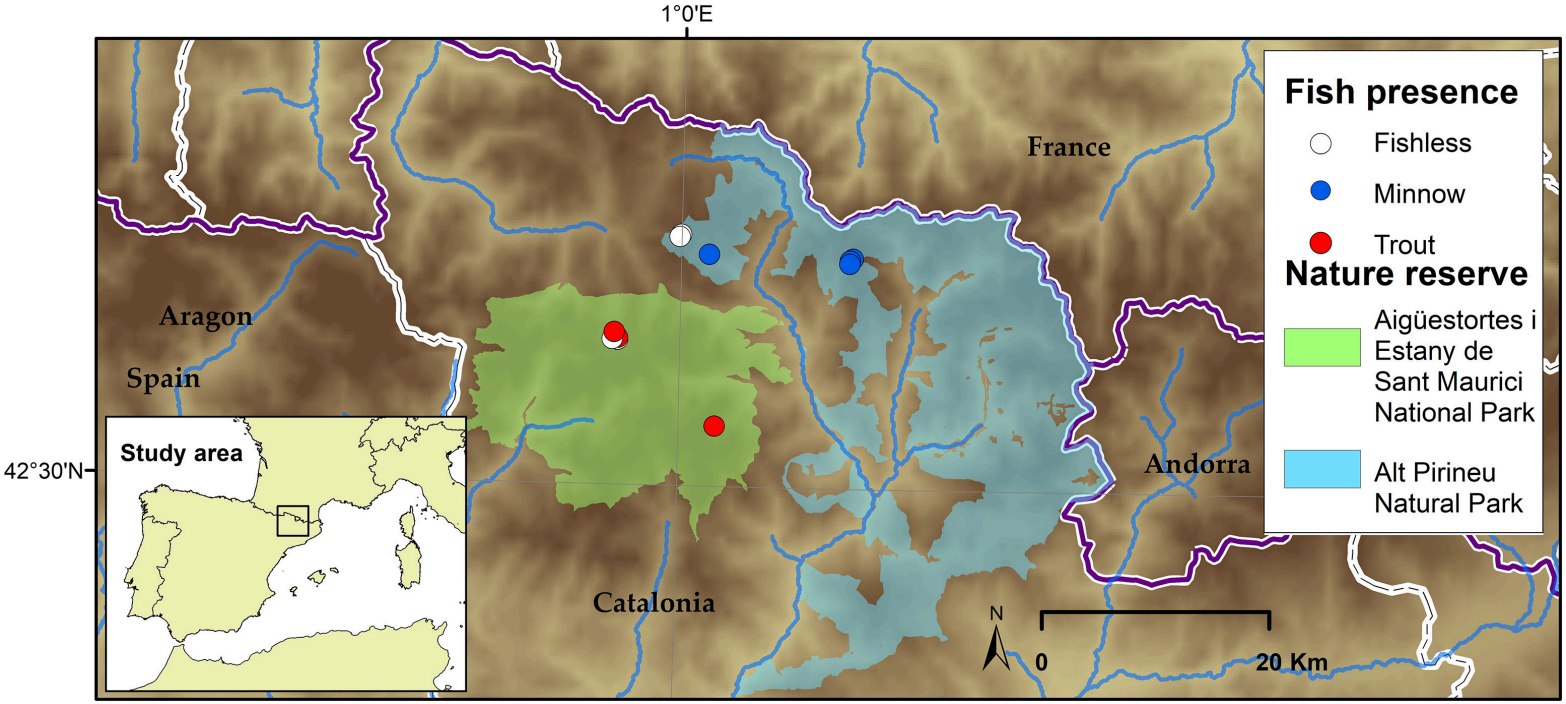
Study site map including the lakes sampled in the Central and Eastern Pyrenees. White color refers to fishless lakes, red as for lakes invaded with trout, and blue as for lakes invaded by dense minnow populations.

A total of 10 Pyrenean alpine lakes encompassing three different scenarios; with trout, with minnows and without fish (Figure 1) and holding *Isoetes lacustris* vegetation were visited in mid-summer during years 2015 and 2016 for an extensive macrophyte population structure and environment (water column and sediment) characterization. The lakes are situated within natural protected areas in the Central Pyrenees. They are relatively shallow (maximum depth close to 10 m) with low surface area (<5 ha), and distributed from 2,188 m a.s.l. to 2,412 m a.s.l. (see Table 1). Densities of non-native fishes range from 5 to 50.25 CPUE for trout and from 4 to 124 CPUE for minnows.

**Table 1:** Physical and morphological descriptors of the lakes studied including macrophyte richness (S).

**Lake**	**X_DEC**	**Y_ DEC**	**h (m)**	**Size (ha)**	**LDepth**	**I_m_ (%)**	**S**	**Fish**	**Catch (ha)**	**Cattle (number m^−1^)**
Bacivèr Superior	0.99956	42.69918	2,326	0.43	1.4	75.60	4	no	4.81	0.16
Bacivèr Inferior	0.99793	42.69729	2,307	0.90	1.5	74.00	5	no	23.77	0.07
Gargolhes 3	0.92792	42.61544	2,272	0.68	3.5	49.66	2	no	26.72	0.27
Gargolhes Superior	0.93358	42.61509	2,305	0.95	6.2	28.94	2	no	4.24	0.16
La Cabana	1.03898	42.54804	2,376	2.33	11.7	7.38	5	trout	168.14	0.05
Plan	0.92959	42.62115	2,188	4.96	12	9.07	4	trout	22.84	0.14
Gargolhes Inferior	0.93354	42.61660	2,299	2.13	9.8	4.44	2	trout	8.23	0.11
Inferior 3 Estanys	1.18105	42.68073	2,411	2.79	9.9	0.71	3	minnow	57.55	0.04
Tres Estanys Mig	1.18413	42.68235	2,412	3.70	10.3	1.26	4	minnow	43.55	0.11
Ventolau	1.18069	42.67825	2,344	2.33	11.7	18.27	1	minnow	28.05	0.26

*Geographic location is provided in the Geographic coordinate system (datum ETR89). Lake altitude (h), lake size, lake maximum depth (LDepth), percentage sub-surface irradiance at meadow depth (I_m_), catchment area (Catch) and number of cattle faces around lake perimeter*.

Underwater light climate was estimated from Secchi disk depth measurements (Z_SD_; m) and estimation of the vertical attenuation coefficient (k_d_; m^−1^) using the relation of Poole and Atkins (1929); a value of 0.2 for K_d_ was used when Secchi depth reached lake bottom. The percentage of subsurface light reaching the depth of the meadows (I_m_; %) was estimated using the equation:

(1) $$\begin{array}{r} {\text{I}}_{\text{m}}=100{\text{e}}^{-\text{Kd}*\text{Zm}} \end{array}$$

Were Z_m_ is the depth in meters where the plants have been sampled. Superficial (upper 5 cm) sediment was collected by a diver by using three PVC of 6 cm in diameter and 30 cm in length sediment cores and the sediment was preserved for analysis. Small sediment traps were deployed within the plant canopy for a period of 2 to 3 weeks at midsummer to measure gross sedimentation within the quillwort populations. The traps consisted of 20.5 ml glass tubes of 1.6 cm diameter by 8.5 cm length attached by groups of 5 to 30 cm bars, and separated 4 cm apart from each other (Gacia and Duarte, 2001). Bars were fixed at about 20 cm above the bottom within the leaf canopy. The structures were deployed and collected by snorkeling. The traps were maintained cold after retrieval until reaching the laboratory. Water for nutrient analyses was collected from the outlet of the lake while the water column chlorophyll samples were collected at the deepest area of the lake at 1.5 times the Z_SD_ or 1 m above the sediment if the Secchi disk arrived to the bottom.

**Figure 2:**
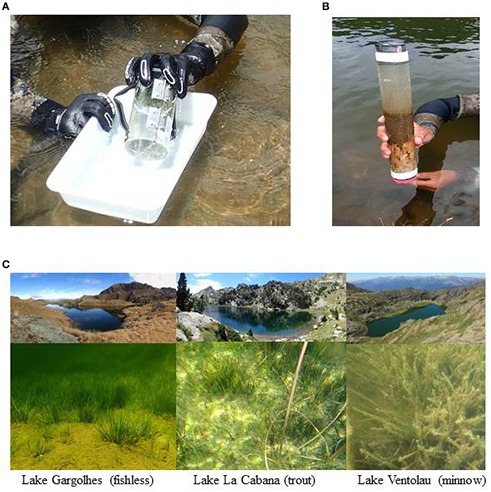
Cylindrical section dredge used to collect quillwort leaves **(A)** and a core of sediment from Tres Estanys **(B**; minnow lake) were different phases of the water-sediment interphase can be distinguished. Lake scenarios under examination **(C)** with an underwater view of the quillwort meadows.

Three samples of the plant community (area of 20 × 20 cm^2^ each one) for plant biomass assessment were collected by a diver at 2 m depth, haphazardly within the quillwort meadow. Three more independent samples of quillwort leaves were collected with a specific cylindrical section dredge (see Figure 2A) that allowed the collection of the leaves and the surrounding water to minimize epiphyte loss after collection; these samples were used to estimate epiphyte load and epiphytic community composition. Cattle impact around the lake was estimated from the counts of faces on a 20 m width area around the lake shoreline.

### Analysis

#### Water and sediment

Water conductivity (Cond), alkalinity (Alk), total nitrogen (TN), dissolved inorganic nitrogen (DIN), total phosphorus (TP) and water column chlorophyll (W_Chl-a) were analyzed following the methods described in Ventura et al. (2000) (Table 2). Organic content of the upper sediment collected with the cores was measured in % as dry weight after ignition. Content of the sediment traps was filtered, frozen and lyophilized to measure the total amount of dry weight deposited. Data was scaled to m^−2^ day^−1^ taking into account the area of the traps (2,010 cm^2^) and the deployment time.

**Table 2:** Biogeochemical variables considered for the studied lakes.

**Lake**	**Cond (μS cm^−1^)**	**Alk (μeq L^−1^)**	**TN (μg L^−1^)**	**DIN (μg L^−1^)**	**TP (μg L^−1^)**	**W_Chla (μg L^−1^)**
Bacivèr Superior	14.03	43.8	298.8	12.3	9.2	1.4
Bacivèr Inferior	9.47	35.3	623.6	35.9	9.5	0.3
Gargolhes 3	19.71	222.6	221.7	29.2	8.4	0.9
Gargolhes Superior	12.23	136.1	143.2	10.1	6.5	1.1
La Cabana	11.6	58.8	194.4	17.1	1.7	0.7
Plan	13.32	148.4	191.6	6.1	5.5	1.1
Gargolhes Inferior	4.94	55.7	186.2	7.9	6.7	3.3
Inferior 3 Estanys	16.43	28.9	269.4	3.4	15.2	7.9
Tres Estanys del Mig	16.49	31	281.4	4.1	16.7	7.2
Ventolau	12.54	71.2	143.6	1.3	18.5	2.5

*Conductivity (Cond), alkalinity (Alk), total nitrogen (TN), dissolved inorganic nitrogen (DIN), total phosphorus (TP) and water column chlorophyll a (W_Chla)*.

#### Plant biomass and epiphytes

Biomass samples -sorted in three different plant parts: leaves, shoots and roots- were dried at 60°C until constant weight. The algae attached to plant leaves collected with the dredge were scraped off using tweezers and collected in a known volume of water. An aliquot of 300 ml was preserved at −20°C for pigment analysis. The plant leaves were kept to calculate leave surface area and biomass dry weight. In one of the lakes (Plan) we could not process the sample from mid-summer and collected epiphyte biomass and pigments in October. This data is not included in the univariate plots but in the PCA on pigment analysis to discuss about the seasonality effect.

#### Pigment analysis of the epiphytes

For pigment analysis, 50–100 mg of freeze-dried material was extracted in 90% acetone with a probe sonicator (Sonopuls GM70 Delft, The Netherlands) (50W, 2 min). The extract was centrifuged (4 min at 3000 rpm, 4°C), filtered through Whatman ANODISC 25 (0.1 μm) and analyzed with ultra-high-performance liquid chromatography (UHPLC). The UHPLC system (Acquity Waters, Milford, MA, USA) was equipped with an UPLC HSS C18 SB column (dimensions: 2.1 × 100 mm; particle size: 1.8 μm) and with PDA (λ: 300–800 nm). The PDA channel was set at 440 nm for pigment detection and quantification. After a sample injection (7.5 μL), the pigments were eluted with a linear gradient from 100% solvent B (51:36:13 methanol:acetonitrile: MilliQ water, v/v/v 0.3 M ammonium acetate) to 75% B and 25% A (70:30 ethyl acetate: acetonitrile, v/v) for 3 min, followed by 0.45 min of isocratic hold at 75% B and 2 min of linear gradient to 100% of solvent A. The initial conditions (100% B) were linearly recovered in 0.65 min. The flow rate was 0.7 ml min^−1^. Pigments were identified by comparison with a library of pigment spectra obtained from extracts of pure algae cultures from the Culture Collection of Algae and Protozoa (CCAP, Oban, Scotland, UK) and pigment standards (DHI Water and Environment, Hørsholm, Denmark). Pigment molecular weight was obtained from the literature (Jeffrey et al., 1997). Marker pigments of cyanobacteria and eukaryotic algal groups were expressed as percentages. Chl-a concentration of epiphytes was expressed as mg cm^−2^ of leaf based on dry weight vs. surface area ratios of the different lakes and scenarios.

#### Carbohydrate content

The carbohydrate concentration in the different carbon storage plant compartments (i.e., leaves, sporangia at the base of the leaves, and corms) was determined by pooling together ten individuals from each lake collected at −2 m depth. Although leaves from different ages (i.e., different position in the rosette) show some variation in total carbohydrate content (Chappuis et al., 2015), all the leaves were pooled together based on the fact that plants were collected at the end of the growing period in October, and thus we measured average carbon storage for the population before winter. Plant compartments were sorted, dried and grinded for further analysis following the method described in Alcoverro et al. (2001) and the extracts read in a spectrophotometer Shimadzu uv-2401 PC. Starch and sucrose were expressed as percentage of dry weight (DW) and were added to obtain the total non-structural carbohydrates (TNC).

#### Statistical analysis

Cumulative distributions of all variables were compared against normal-distribution function using the Shapiro-Wilk test. Non-normal variables (*p* < 0.05) were square root or log transformed to better distribute the data. Principal component analysis (PCA) was used to summarize morphometric and physicochemical descriptors of the water column. Physicochemical structure complexity was reduced by projecting samples on the first axis and second axis (PC1 and PC2). Spearman rank-order correlation coefficients were determined for pairwise comparisons of physicochemical (dataset values and PC1, PC2 scores), benthic and meadow variables at *P* < 0.05. We used Nested ANOVAs and Tukey's HSD *post-hoc* test to determine whether variables exhibited significant variation between scenarios (i.e., fishless lakes, with trout or with minnows). Analyses were performed using PRIMER 6.0 (for PCA) and the Statistica software package (StatSoft, Tulsa, OK, USA).

## Results

### Lakes environment

**Figure 3:**
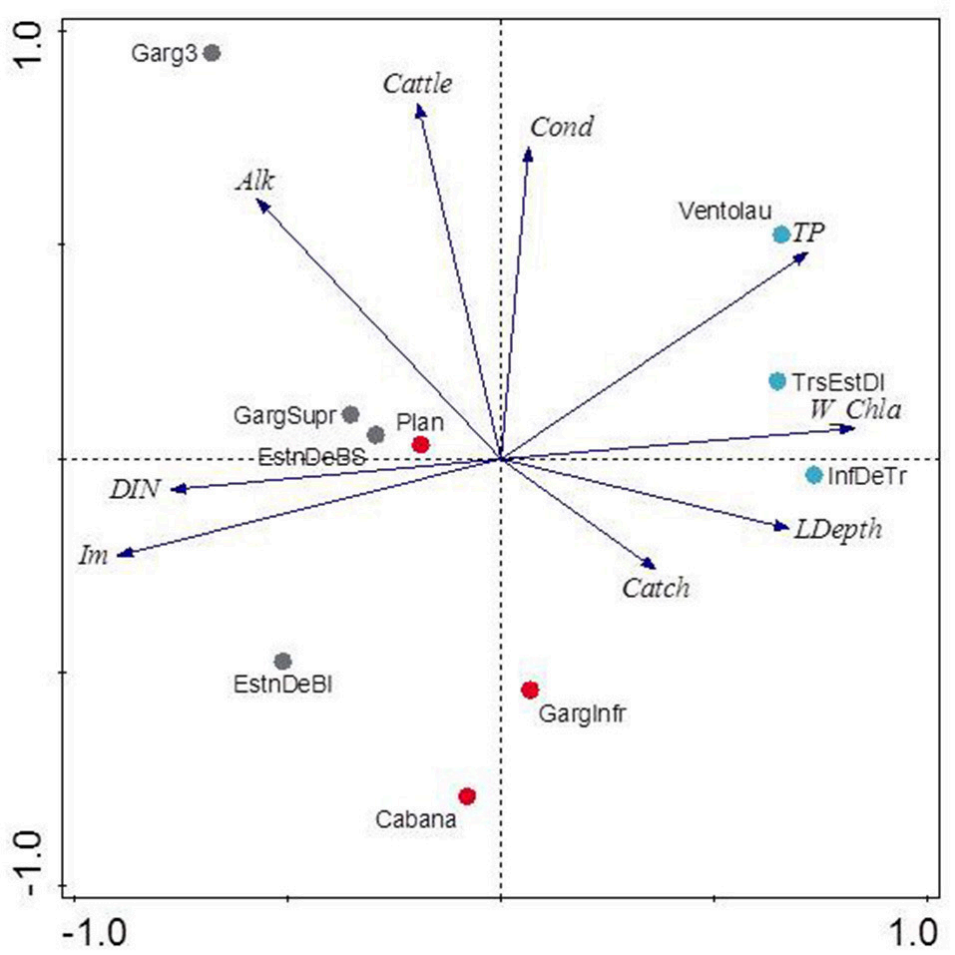
Principal Component Analysis (PCA) of the water column physico-chemistry and morphological parameters from ten lakes. Gray symbols are fishless lakes, red as for lakes invaded with trout and blue as for lakes invaded with dense minnow populations. Percentage of the variance explained by the first two axes is 39.9% and 22.2%, respectively. See Table 1 for variables abbreviation and Annex 1 Supplementary Materials for the score values. Lake name abbreviations are as follow: Bacivèr Superior (EstnDeBS), Bacivèr Inferior (EstnDeBI), Gargolhes 3 (Garg3), Gargolhes Superior (GargSupr), La Cabana (Cabana), Plan, Gargolhes Inferior (GargInfr), Inferior 3 Estanys (InfDeTr), Tres Estanys Mig (TrsEstDI), Ventolau.

**Figure 4:**
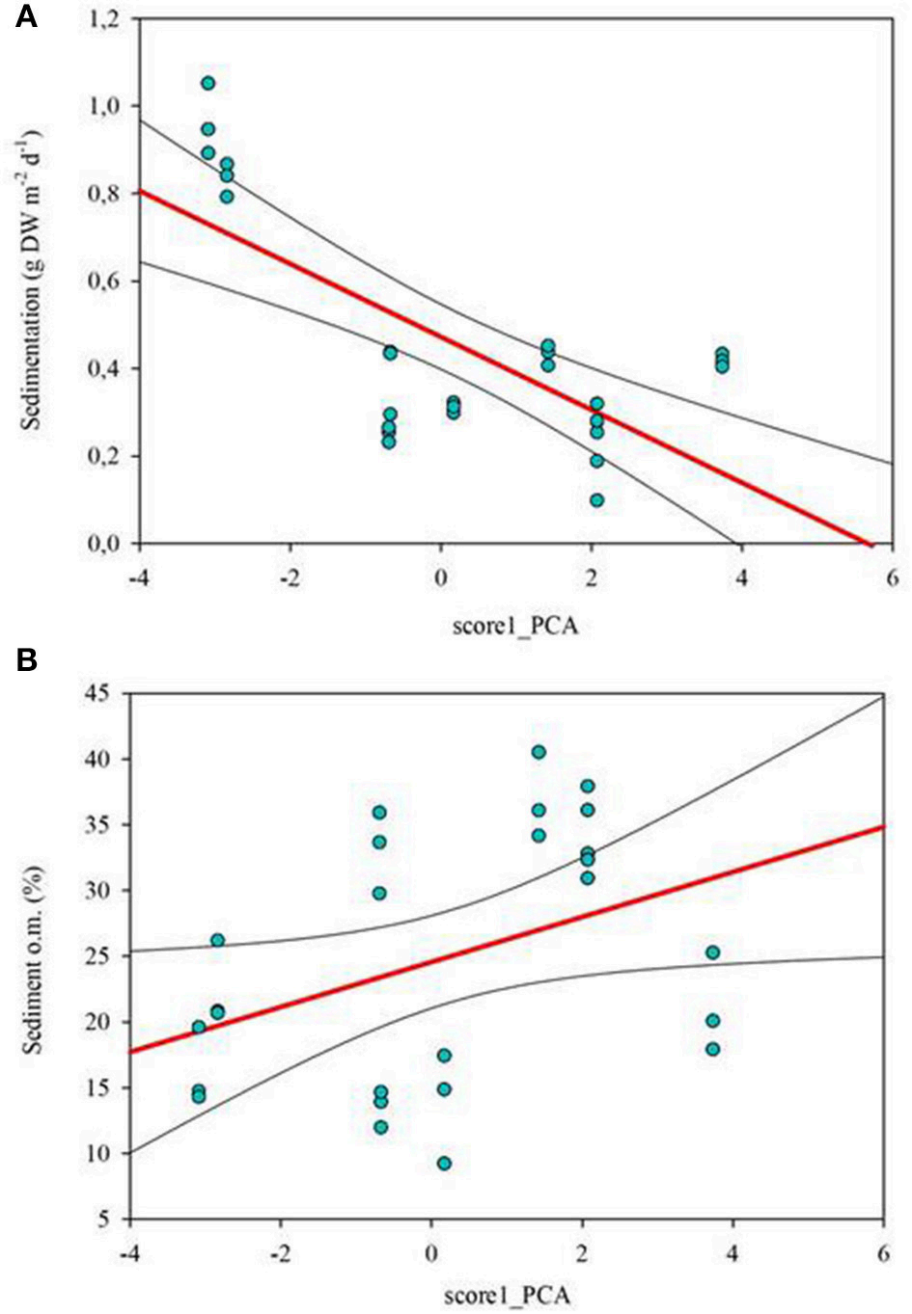
Correlations between physicochemical variation of the water column measured by PC1 score from Figure 3 and sedimentation rates [g dry weight (DW) m^−2^ d^−1^; **(A)]** and sediment organic content (in %; **B**). PC1 explained 39.9% of the variability of the samples and it was mainly accounted by sub-superficial percentage of light in the meadow (Im), water column Chl-a (W_Chl-a) and total phosphorus. Correlations are given as Spearman's rank-order correlations (rho) in Table 3. Significant values are set at *p* < 0.05.

**Figure 5:**
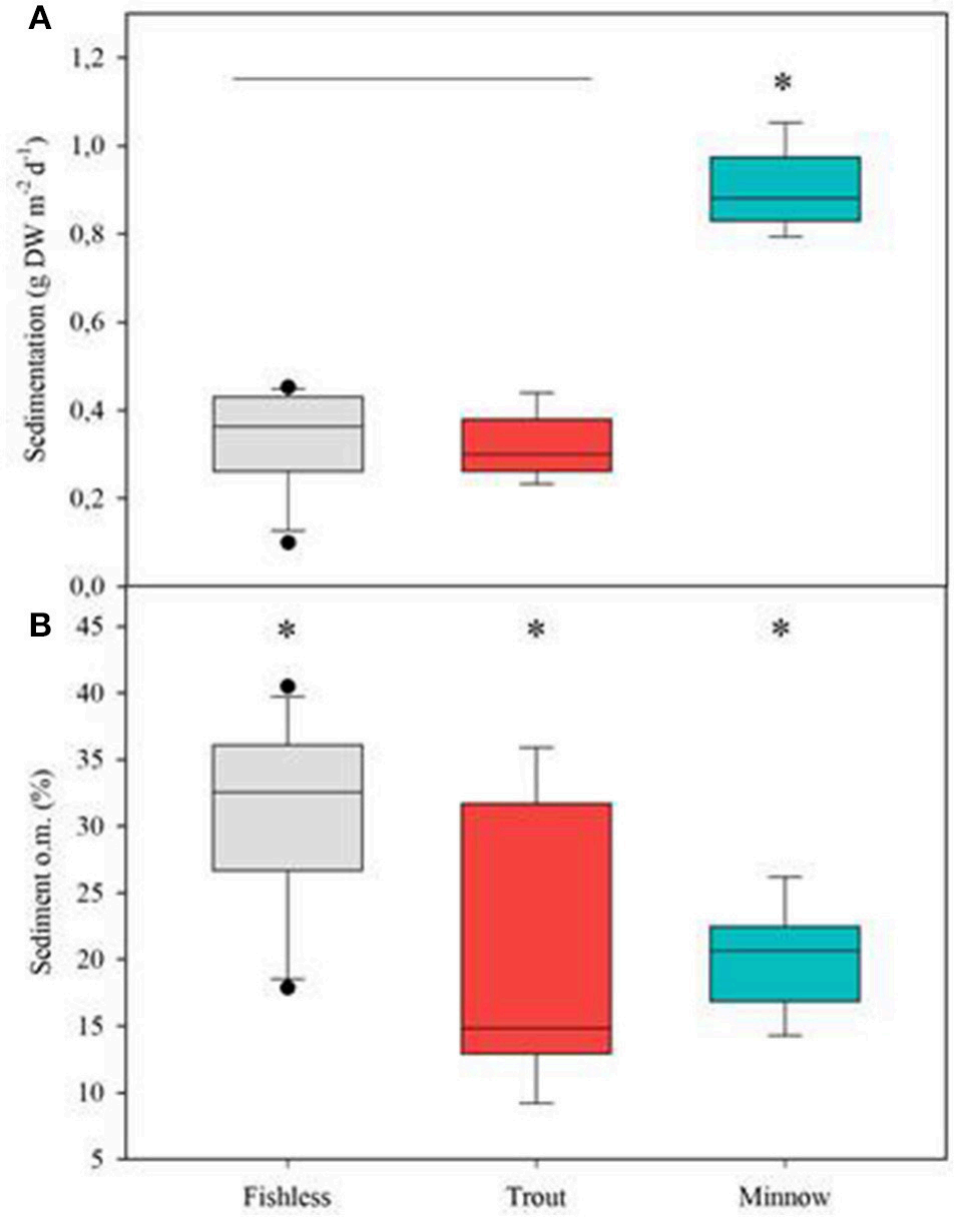
Box plots for **(A)** total sedimentation, **(B)** sediment organic content (o.m.) in % in relation to the different scenarios: fishless, trout and minnows. Differences in the mean values are tested following the ANOVA model; var (x) = Scenario + Lake (Scenario) + error; significant differences are set at *p* < 0.01. Asterisks denote significantly different from the rest and the horizontal line indicates groups not significantly different.

Those variables accounting for the maximal physicochemical and morphological variability among the studied lakes were light irradiance at the meadows (I_m_) and dissolved inorganic nitrogen (DIN), opposite to water column Chl-a and total phosphorus (PC1, 39.9 % of the variability), and cattle pressure, conductivity and alkalinity (PC2, 22.2% of the variability; Figure 3; Annex 1 in Supplementary Materials). This first ordination is related to planktonic primary production promoted by high phosphorus that results in low water transparency. The second ordination is all related to catchment processes such as cattle pressure, conductivity and alkalinity (Figure 3; Annex 1 Supplementary Materials). The projection of the lakes under the different scenarios (i.e., fishless, trout and minnows) onto the first PC1 shows a clear segregation of the lakes per scenarios, so that lakes with minnows pack together and appear linked to low water transparency and high phosphorus, while fishless lakes show a pattern of non-disturbed waters with high water transparency and DIN. Trout lakes occupy an intermediate position. Lake ordination across the PC1 can thus be used as a proxy of the relation between the scenarios and the water column environment; it significantly correlates with descriptors of the benthic environment such as total sedimentation and organic content of the sediment (Figures 4A,B; Table 3) thus proving a bentho-pelagic coupling in the studied systems. Indeed total sedimentation rates differed for some of the scenarios and were up to 2.5 times higher in the presence of minnows compared to lakes with trout and fishless (Figure 5A). The organic content of the solid phase of the sediment significantly differed and was lower in the presence of fishes (both minnows and trout) compared to fishless lakes (Figure 5B). However, there was a high liquid-floccule organic phase in the presence of fishes (Figure 2).

**Table 3:** Correlation coefficients (R) between descriptors of the benthic environment and scores of the PCA.

**Benthic variables**	**PC1**	**PC2**
Sedimentation (g DW m^−2^ d^−1^)	0.711^**^	–
Upper Sediment (% om)	0.401^*^	–
log Total Biomass (g DW m^−2^)	0.405^*^	0.509^**^
log Quoc (Above vs. Below)	0.742^**^	–
log Epiphytic Chla mg.cm^−2^	0.755^**^	–
Log ∑ Cyano	0.850^*^	–
Log ∑ Chloro	0.837^*^	–
Log ∑ Diatom	–	–

n = 10 studied cases for meadow variables and n = 9 for epiphytic algal group composition. Only significant correlations at p < 0.05* and p < 0.0005** are shown.

### Quillwort populations

#### Plant biomass

**Figure 6:**
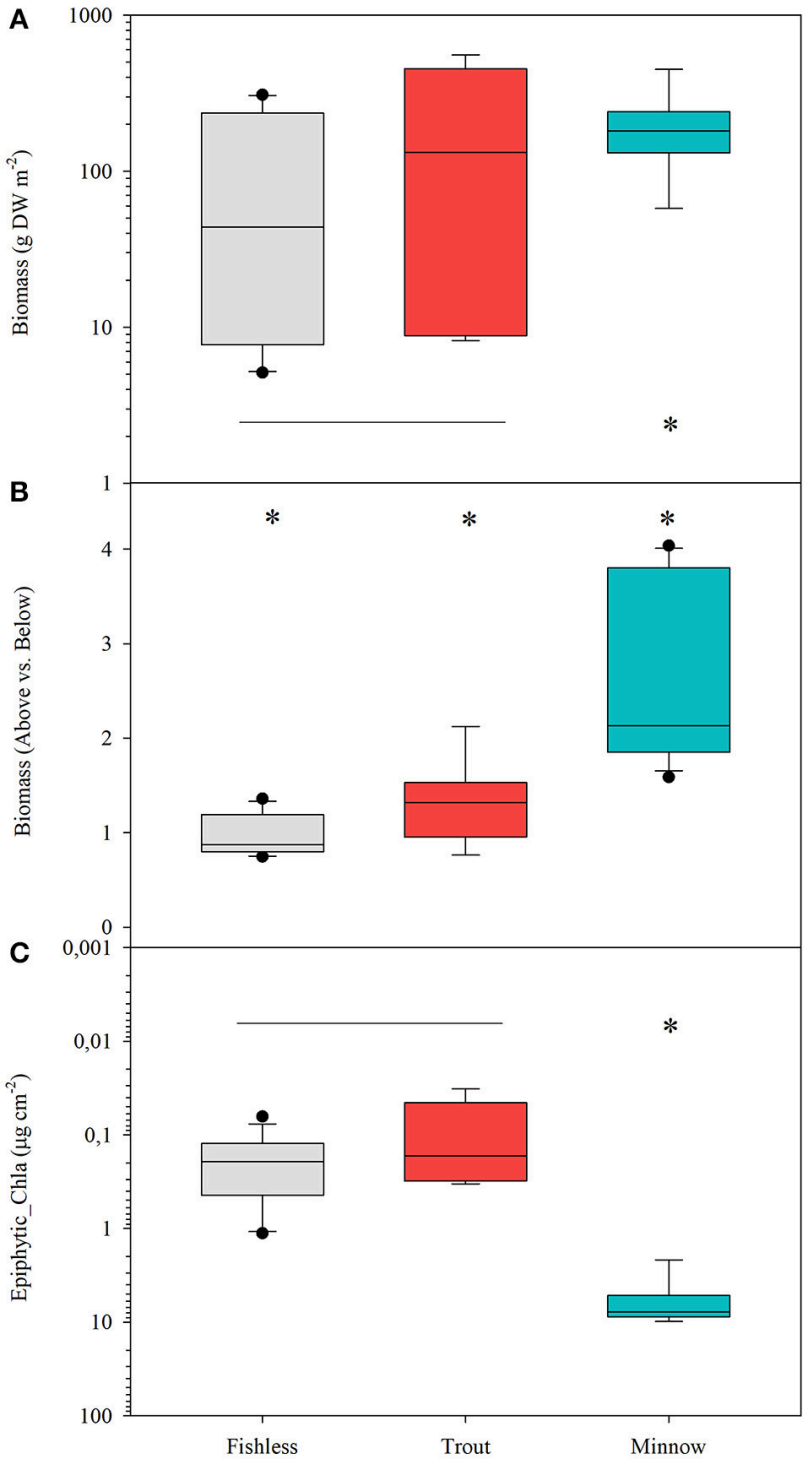
Box plots for **(A)** total biomass of quillwort meadows in log DW m^−2^, **(B)** quotient above vs. belowground biomass and **(C)** load of epiphytes expressed as μg chl-a cm^−2^ of leaf in 10 lakes under the different scenarios; fishless, with tout and with minnows. Differences in the mean values are tested following the ANOVA model; var (x) = Scenario + Lake (Scenario) + error; significant differences are set at *p* < 0.01. Asterisks denote significantly different from the rest and the horizontal line indicates groups not significantly different.

Quillwort population biomass per unit area significantly differed among lakes (Table 4). The highest values were found in La Cabana (average 0.65 ± 0.14 g DW 400 cm^−2^) and the lowest in Gargolhes Superior (average 0.01 ± 0.001 g DW 400 cm^−2^). Total plant biomass shows a higher correlation with PC2 (conductivity) than with PC1 (irradiance at meadow depth and water column Chl-a; Table 3). Therefore, the community of non-native fishes had no consistent effect on quillwort biomass (Figure 6A; Table 3). Total biomass did not distribute equally within the plant compartments and the quotient of Above vs. Belowground biomass differed among lakes and scenarios (Table 4) being significantly higher in the presence of minnows, intermediate in trout lakes and lower in fishless lakes (Figure 6B). This quotient highly correlated to the amount of material deposited in the traps (*R* = 0.380; *p* < 0.05) suggesting a phenotypic response of the shoots by increasing aboveground biomass in response to increased sedimentation.

**Table 4:** Summary of the Nested ANOVAs [var = Scenario + Lake (Scenario) + error] of quillwort meadow biomass, areal vs. belowground biomass and Chl-a of the epiphytic community.

	**SS**	**DF**	**MS**	**F**	**p**	**Comparison**
**QUILLWORT BIOMASS**						
Intercept	4.108	1	4.108	190.0	<0.001	
Scenario	3.050	2	1.525	70.5	<0.001	Min>Nat>Trout
Lake (Scenario)	10.918	7	1.559	72.1	<0.001	
Error	0.432	20	0.021			
**AREAL vs. BELOWGROUND BIOMASS**						
Intercept	10.885	1	10.885	1, 585.6	<0.001	
Scenario	1.231	2	0.615	89.7	<0.001	Min>Trout>Nat
Lake(Scenario)	0.222	7	0.031	4.6	<0.005	
Error	0.137	20	0.006			
**EPIPHYTIC Chl-a**						
Intercept	166.565	1	166.565	2, 310.8	<0.001	
Scenario	19.977	2	9.988	138.5	<0.001	Min>Trout, Nat
Lake(Scenario)	5.914	8	0.739	10.2	<0.001	
Error	1.585	22	0.072			

*Data were log transformed. Tukey HSD post-hoc comparison tests were run when significant differences were detected among scenarios (Nat = fishless, trout, Min = minnows) in order to know which ones differed. Abbreviations: SS, Sum of squares; DF, degrees of freedom; MS, Mean square; F value, F test statistic*.

#### Epiphyte biomass and community composition based on pigments

**Figure 7:**
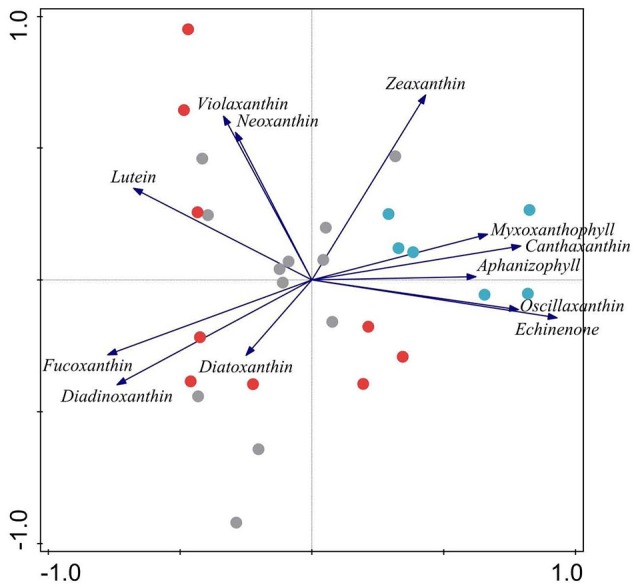
Principal component analysis of the epiphytic algal groups pigment composition in %. Gray lakes are for fishless lakes, red as for lakes invaded with trout and blue as for lakes invaded with dense minnow populations. Percentage of the variance in marker pigment composition explained by the first two axes variance is 61%.

Chl-a of *I. lacustris* epiphytes, a surrogate of epiphytic algal biomass, was up to eight times higher in the presence of the introduced minnows than in lakes with trout or fishless (Figure 6C). Ordination of the samples of the epiphytic community based on pigments showed that the first two components of the PCA captured 73% of the variance in marker pigment composition (Figure 7; Annex 2 Supplementary Materials). The algal group composition in lakes with minnows was markedly different from the other lake types and dominated by cyanobacteria (myxoxanthophyll, aphanizophyll, oscillaxanthin, echinenone, and zeaxanthin). Chlorophytes (lutein, neoxanthin, violaxanthin, and zeaxanthin) and diatom marker pigments (fucoxanthin, diadinoxanthin, and diatoxanthin) were present in lakes with trout or fishless. Chlorophytes and cyanobacteria positively correlated with the scores of the water column PCA ordination (Table 3).

#### Carbohydrate content

**Figure 8:**
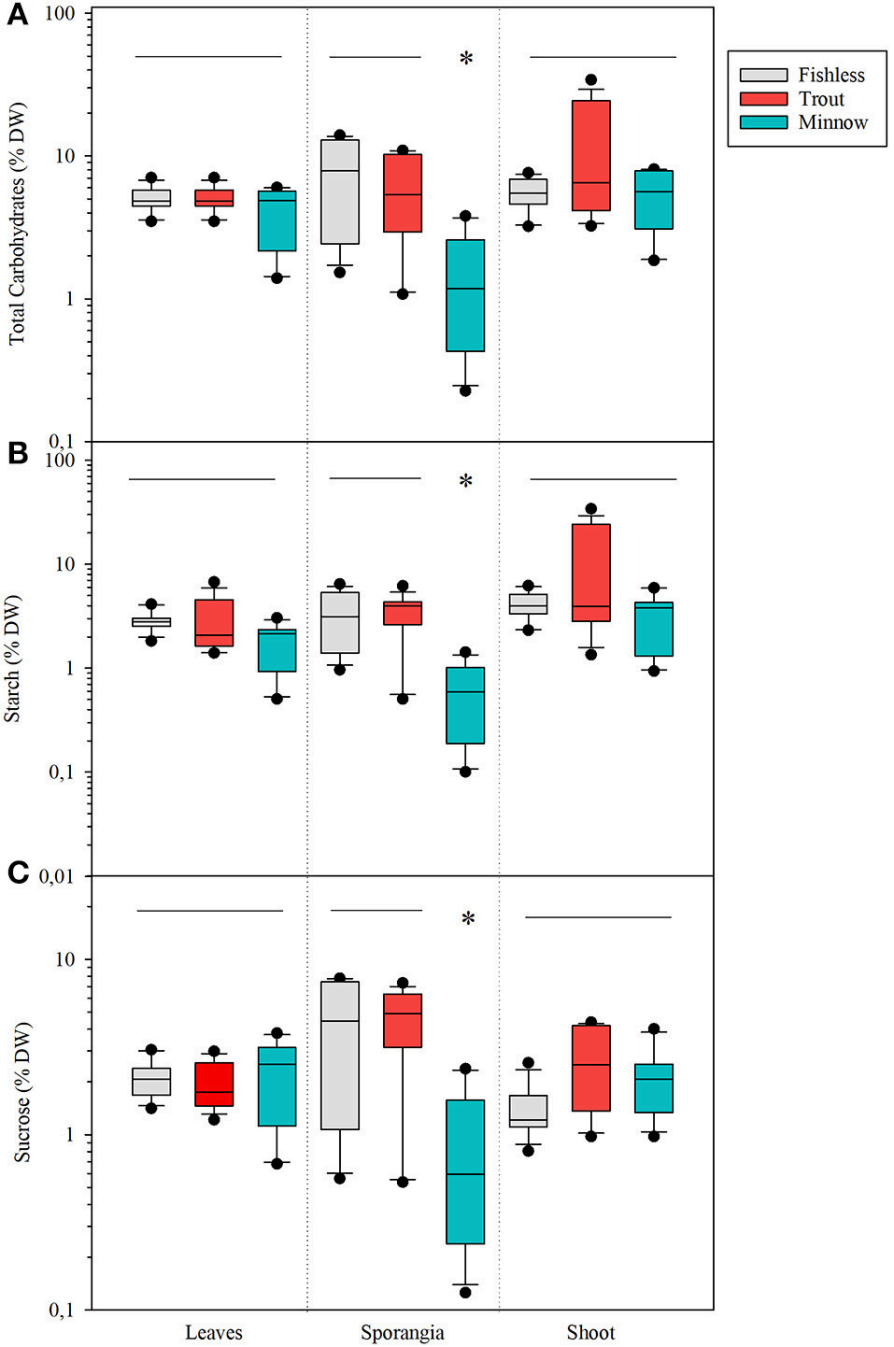
Box plots for **(A)** total carbohydrate content as % DW, **(B)** total starch content as % DW, and **(C)** total sucrose content as % DW in quillwort sorted by plant compartments (leaves, sporangia and corms) under the different scenarios: fishless in gray, with tout in red, and with minnows in blue. Significant differences are set at *p* < 0.01. Asterisks denote significantly different from the rest and the horizontal line indicates groups not significantly different.

Total content of carbohydrates significantly varied among scenarios, lakes and compartments (Table 5). Shoots of lakes with minnows had average total carbohydrate content significantly lower than shoots from fishless or with trout (Table 5). Sporangia were the compartment showing the highest variation (range from 13.9 to 0.2 % DW), followed by leaves (range 7.0 to 1.4 % DW) and corms (range from 6.8 to 0.5 % DW) that showed similar values. Total carbohydrates in plant compartments interacted with lake scenarios because the populations in the natural and trout lakes had significantly much larger concentration of carbohydrates in the sporangia than in the other compartments, while in the presence of minnows the tendency was opposite (i.e., significantly lower concentrations; Figure 8A). The same pattern was found for the concentration of starch and soluble carbohydrates (Figures 8B,C) with total values ranging from 6.4 to 0.1% DW for starch and from 7.7 to 0.1% DW for soluble carbohydrates, respectively.

**Table 5:** Summary of the Nested ANOVAs [var = Scenario + Lake (Scenario) + Part (Lake ^*^ Scenario) + error] of *Isoetes lacustris* carbohydrate storage (total, sucrose and starch) under the different lake scenarios (Nat = fishless, trout, Min = minnows).

	**SS**	**DF**	**MS**	***F***	***p***	
**TOTAL CARBOHYDRATES (%)**						
Intercept	43.04	1	43.04	6, 066.7	<0.001	
Scenarios	1.97	2	0.99	138.9	<0.001	Min < Trout, Nat
Lake(Scenario)	3.19	9	0.35	50.0	<0.001	
Part(Lake^*^Scenario)	8.64	24	0.36	50.7	<0.001	Spor ≠ Corm, leaves
Error	0.55	78	0.01			
**STARCH (%)**						
Intercept	14.75	1	14.75	1, 721.0	<0.001	
Scenarios	3.54	2	1.77	206.8	<0.001	Min < Trout, Nat
Lake(Scenario)	5.91	9	0.66	76.6	<0.001	
Part(Lake^*^Scenario)	9.16	24	0.38	44.5	<0.001	Spor ≠ Corm, leaves
Error	0.67	78	0.01			
**SUCROSE (%)**						
Intercept	7.88	1	7.88	949.5	<0.001	
Scenarios	1.18	2	0.59	70.9	<0.001	Min < Trout, Nat
Lake(Scenario)	2.96	9	0.33	39.6	<0.001	
Part(Lake^*^Scenario)	8.47	24	0.35	42.5	<0.001	Spor ≠ Corm, leaves
Error	0.65	78	0.01			

*Different plant parts (Part) were analyzed: leaves, sporangia (base of the leaves) and corms. Data as % dry weight were Log transformed. Abbreviations: SS, Sum of squares; DF, degrees of freedom; MS, Mean square; F value, F test statistic*.

## Discussion

There is an important gradient in the physico-chemistry of the studied lakes with records of light water attenuation and water column total phosphorus well above the maximum recorded to date in the area of study (Camarero et al., 2009; Catalan et al., 2009). In fact, the average values of total phosphorus encountered in minnow lakes correspond to that usually found in mesotrophic lakes. The benthic environment also showed that total sediment deposition was much higher in minnow lakes.

Total macrophyte biomass per unit area was marginally affected by the presence of non-native fishes but the biomass allocation within the shoot varied substantially among scenarios. Quillworts show very high plasticity of the photosynthetic tissues (Hickey, 1986; Chappuis et al., 2015) with increasing above against belowground biomass under conditions of low light availability. In minnow lakes, high water column light attenuation levels and high epiphytic biomass were coupled to an increase of up to 6 times the biomass of quillwort leaves at expenses of the belowground compartments (shoots and roots) and reproductive effort (sporangia). These results do not seem to indicate that quillwort are below light compensation levels in minnow lakes, which would cause shoot mortality within two months (Rørslett and Johansen, 1996). However, reduced biomass and much lower total and soluble carbohydrate concentration of sporangia from minnow lakes suggest light stress impairing reproductive performance (Vöge, 1997). Indeed, quillwort can only reproduce sexually and need large sporangia with mature spores for successful reproduction (Farmer and Spence, 1986). Our results are thus a sign that quillwort meadows of lakes invaded by dense minnow populations are threatened by limited growth and expansion in comparison to populations from lakes invaded by trout or fishless.

The signs of stress in the plants shown in minnow lakes (large biomass allocated to the aboveground compartment and reduced carbohydrate concentration in the sporangia) do not explain macrophyte mortality for carbon unbalance but we have recently seen mats of alive quillwort floating in three of the lakes invaded by minnows: Tres Estanys de Dalt, Inferior 3 Estanys and Ventolau. This is compatible with quillwort shoots exceeding the stable limits of its negative buoyancy, as described in *Littorella uniflora* and *Lobelia dortmanna*, and therefore regressing in impacted lakes by episodes of uprooting (Røelofs et al., 1994; Spierenburg et al., 2013). The quillwort biomass allocation ratio measured in minnow lakes are up to 2.5 times higher than under natural conditions and well above the risk of uprooting described for other isoetids (Røelofs et al., 1994). This is usually associated to processes not only of light impairment but of decreasing sediment compactness that occurs along with eutrophication (Spierenburg et al., 2013; Lucassen et al., 2016).

The biomass of the quillwort's epiphytic community was very high and dominated by cyanobacteria in minnow lakes and low and composed of chlorophytes and diatoms in fishless and trout lakes. Cyanobacterial blooms are typical from eutrophic systems (Reynolds, 1998) and various explanations of cyanobacterial dominance at high nutrient levels have been proposed, e.g., changes in the availability of phosphorus and the relative availability of P and N (DIN/TP ratio), light conditions, pH/CO_2_ conditions, and grazing (e.g., Smith, 1986; Shapiro, 1990). Although we cannot point to a single explanation, it seems clear that reduced light levels and increased P availability are at least partly responsible for the shift from chlorophytes and diatoms to cyanobacteria in the epiphytic community of quillworts from minnow lakes.

Altogether it seems that in minnow lakes high densities of those small fish exert a strong predation on zooplankton (Schabetsberger et al., 1995) and zoobenthos (V. Osorio Pers. Comm.) along with sediment resuspension and nutrient release (Hesthagen et al., 1992; Schindler et al., 2001). Altogether results in an increase in water column chlorophyll and potentiates epiphytes overgrow. Enhanced nutrients seem to be responsible for cyanobacterial blooms epiphytizing quillwort leaves and low light availability altogether is responsible for the increase in the aboveground: belowground biomass ratios in quillworts (Chappuis et al., 2015), that can end up unrooted when coupled to decreased sediment compactness. We predict that those fragile former soft-water oligotrophic lakes now infested with minnows might end up with the disappearance of quillwort populations.

Finally, we have to point out that the restoration of these lakes would be hard to accomplish. Isoetid recovery does not only require minnows' removal but also a decrease in sediment organic matter and an enhancement of quillwort recruitment (Pulido et al., 2011, 2012; Riera et al., 2017). Moreover, the restoration of quillwort populations once the natural environmental characteristics of the lake are recovered, seem to proceed very slowly and probably encompass one century or more (Riera et al., 2017). Hence, minnows represent a major threat to underwater quillwort vegetation and to the overall ecological integrity of shallow lakes in the Pyrenees. Trout, at certain densities may also have an impact on epiphyte development because they feed on grazers, but the data reported here is not supporting such a negative effect as minnows do. Last, it is essential to unveil how population dynamics of minnows, but also trout, evolve through time, including their interactions. Without this information we will not be able to make appropriate diagnosis on how these non-native species interact with macrophyte meadows maintenance in the medium to the long term, and thus the chances of invaded lakes to reach hysteresis or collapse (Scheffer and Jeppesen, 2007). Given the strong signs of stress on quillwort populations detected here and the ecosystem deterioration registered, and since soft-water oligotrophic lakes with quillwort vegetation are ecosystems protected under the Habitats Directive, some actions need to be urgently implemented before the scenario gets worse.

## Author contributions

EG contributed to study design, field work, sample processing, student formation, data processing and wrote the ms; TB contributed to study design, field work, sample processing, data processing and helped in writing parts of the ms; NB-M contributed to field work, sample processing, data treatment and drafting; IS contributed largely to the field work, sample processing, and data treatment including plots; EB and MV contributed to study design, field work, sample processing, and help in ms writing.

### Conflict of interest statement

The authors declare that the research was conducted in the absence of any commercial or financial relationships that could be construed as a potential conflict of interest.
