# Supplementary material for: Non-native Minnows Threaten Quillwort Populations in High Mountain Shallow Lakes
Source: Front Plant Sci. 2018 Mar 22;9:329. doi: 10.3389/fpls.2018.00329 (PMC5874306; doi:10.3389/fpls.2018.00329)
Supplement: Supplementary file 1 [file Table1.pdf]

Annex 1. Coefficients of the linear combination of variables composing the first 2 principal component scores of PCA shown in Fig 3.

Variable	PC1	PC2
LDepth (m)	0,675	-0,163
Cond (µS)	0,064	0,730
Alk (µeq L^{-1})	-0,573	0,607
DIN (µg L^{-1})	-0,777	-0,073
TP (µg L^{-1})	0,719	0,483
I_m	-0,900	-0,227
W_Chla (µg L^{-1})	0,830	0,071
Total catchment (ha)	0,362	-0,259
Bestiar (nºexcrem m^{-1})	-0,196	0,832

Annex 2. Coefficients of the linear combination of variables composing the first 2 principal component scores of PCA shown in Fig 7.

Variable	PC1	PC2
Diadinoxanthin	-0,740	-0,398
Lutein	-0,677	0,347
Zeaxanthin	0,431	0,702
Canthaxanthin	0,792	0,129
Diatoxanthin	-0,250	-0,286
Myxoxanthophyll	0,667	0,173
Aphanizophyll	0,623	0,012
Echinenone	0,930	-0,144
Fucoxanthin	-0,775	-0,284
Violaxanthin	-0,335	0,621
Oscillaxanthin	0,784	-0,113
Neoxanthin	-0,290	0,560
